# Compositional Differences and Similarities between Typical Chinese Baijiu and Western Liquor as Revealed by Mass Spectrometry-Based Metabolomics

**DOI:** 10.3390/metabo9010002

**Published:** 2018-12-21

**Authors:** Cheng Fang, Hai Du, Wei Jia, Yan Xu

**Affiliations:** 1 State Key Laboratory of Food Science and Technology, School of Biotechnology, Jiangnan University, Wuxi 214122, China; playfang1989@hotmail.com (C.F.); duhai88@126.com (H.D.); 2 University of Hawaii Cancer Center, Honolulu, HI 96813, USA

**Keywords:** distilled liquor, metabolomics, volatile compounds, HS-SPME-GC-TOFMS, non-volatile compounds, GC-TOFMS

## Abstract

Distilled liquors are important products, both culturally and economically. Chemically, as a complex mixture, distilled liquor comprises various chemical compounds in addition to ethanol. However, the chemical components of distilled liquors are still insufficiently understood and compositional differences and similarities of distilled liquors from different cultures have never been compared. For the first time, both volatile organic compounds (VOCs) and non-VOCs in distilled liquors were profiled using mass spectrometry-based metabolomic approaches. A total of 879 VOCs and 268 non-VOCs were detected in 24 distilled liquors including six typical Chinese baijiu and 18 typical Western liquors. Principal component analysis and a correlation network revealed important insights into the compositional differences and similarities of the distilled liquors that were assessed. Ethyl esters, a few benzene derivatives, and alcohols were shared by most distilled liquors assessed, suggesting their important contribution to the common flavor and mouthfeel of distilled liquors. Sugars and esters formed by fatty alcohol differ significantly between the assessed Chinese baijiu and Western liquors, and are potential marker compounds that could be used for their discrimination. Factors contributing to the differences in chemical composition are proposed. Our results improve our understanding of the chemical components of distilled liquors, which may contribute to more rigorous quality control of alcoholic beverages.

## 1. Introduction

Alcoholic beverages have been a part of human civilization for millennia and they are an indispensable aspect of human culture [1]. During the development of civilization, various distilled liquors were created around the world, including whisky, brandy, rum, vodka, and baijiu (also known as Chinese liquor). Today, distilled liquors are high value products, both commercially and culturally [2], and contribute significantly to total alcohol consumption.

Chemically, distilled liquor is a complex mixture of water, ethanol and several thousands of chemical compounds [3], which contribute to the unique flavor and mouthfeel of each type of liquor. Routine chemical analysis of distilled liquors was conducted using headspace solid phase microextraction (HS-SPME), coupled with gas chromatography–mass spectrometry (GC–MS). This method was proven to be very useful in analyzing the volatile organic compounds (VOCs) that contribute to the flavor of distilled liquors, such as brandy [4], rum [5], whisky [6], vodka [7], and baijiu [8]. However, the chemical components of distilled liquors are still poorly understood, because non-VOCs, which often influence the overall flavor volatility and perception, also exist in distilled liquors [9]. For instance, we recently detected lichenysin (a macro-molecular cyclic lipopeptide) in baijiu [9,10], and caramel is often manually added into aged whisky and brandy [2,11]. The chemical compounds in distilled liquors from different cultures may vary given the use of different raw materials, production processes, and the maturation conditions. For example, most Western liquors are fermented by yeasts, which significantly differs from baijiu, whose fermentation is driven by various microorganisms (bacteria, yeasts, and filamentous fungi) [3]. In addition, baijiu is produced through solid-state fermentation and distillation, whereas Western liquors are produced by submerged fermentation and distillation. Hence, it is reasonable to deduce that the composition of Chinese baijiu and Western liquor is different. However, to the best of our knowledge, these compositional differences and similarities have never been compared.

Metabolomics, as a holistic and powerful approach, may be suitable for analyzing the complete chemical composition of distilled liquors. Metabolomics focuses on the comprehensive measurement of large-scale small molecular metabolites in complex biological and abiotic samples and is the third most commonly employed omics technology, after genomics and proteomics [12,13]. Powered by the unparalleled sensitivity and specificity, high resolution and wide dynamic range of MS [14], automated sample processing, and advanced metabolomic protocols, high-throughput GC–MS is a reliable tool for chemical profiling, and has been extensively applied in the analyses of components from foods and beverages [15,16]. Thus, metabolomics is ideally positioned for the metabolite profiling of distilled liquors. We could also establish more rigorous quality control of distilled liquors through metabolomics. For example, Chinese baijiu is produced through mixed culture fermentation, solid-state fermentation, and simultaneous saccharification and fermentation. The nature of Chinese baijiu production makes it susceptible to environmental factors. Our previous studies revealed that the fermentation process of Chinese light-aroma-type liquor was at risk of contamination with *Streptomyces*. *Streptomyces* is a producer of geosmin, which is known for having a distinct earthy flavor and affecting the quality of Chinese liquor [17,18,19,20]. 

This study aimed to comprehensively analyze both VOCs and non-VOCs in selected distilled liquors, providing chemical characterization for future investigation into the impact of these alcoholic beverages on human health. We compared the chemical composition of liquors from different cultures, such as several Chinese baijius and Western liquors, which could potentially help us to understand the factors that cause the compositional differences in liquors from different cultures. Due to the different compounds that may influence the flavor and taste, or even the safety of liquors (especially for Chinese baijiu, which is produced under natural susceptible conditions), our analysis may also help to improve the mouthfeel and quality of Chinese baijiu. Our study may contribute to the establishment of more rigorous controls on the quality and flavor of distilled liquor and may help us to understand the relationship between the metabolites derived from complex microflora and food safety and nutrition. 

## 2. Results

### 2.1. VOCs Detected by HS-SPME-GC-TOFMS

**Figure 1:**
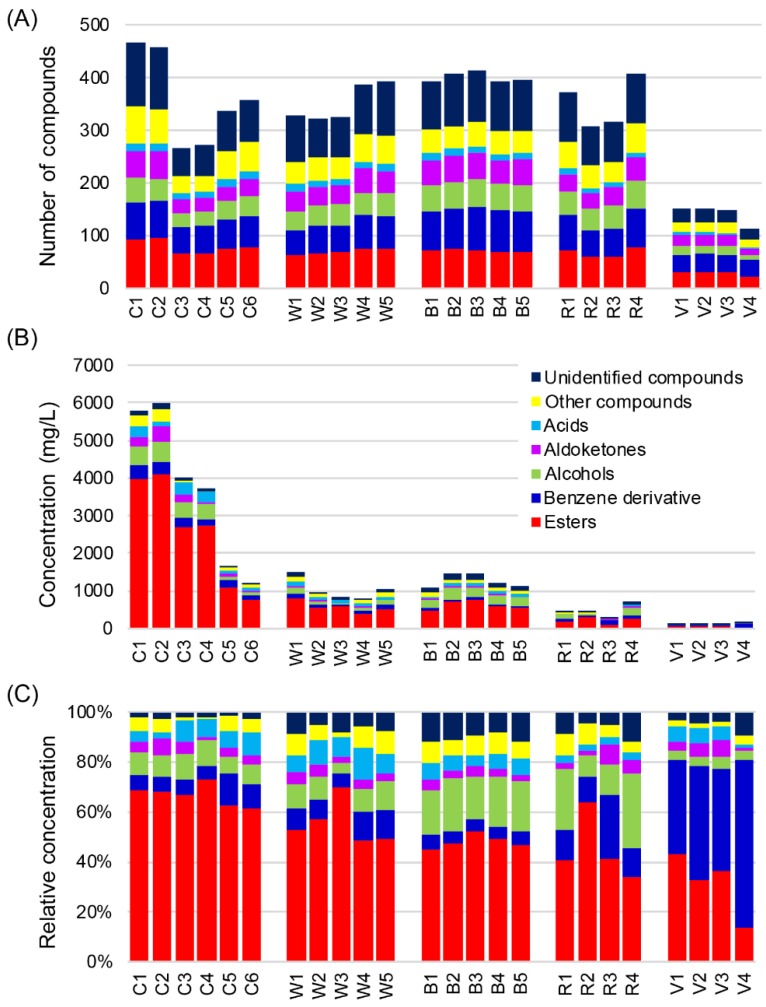
Volatile organic compounds (VOCs) in distilled liquors detected by HS-SPME-GC-TOFMS. (**A**) Number of compounds in major classes of VOCs in 24 representative distilled liquors. (**B**) Contents of major classes of VOCs. (**C**) Relative concentration of major classes of non-VOCs. Abbreviations: C1 and C2, Chinese soy sauce aroma type liquor 1 and 2, respectively; C3 and C4, Chinese strong aroma type liquor 3 and 4, respectively; C5 and C6, Chinese light aroma type liquor 5 and 6, respectively; W1–W5, Whisky 1–5; B1–B5, Brandy 1–5; R1–R4, Rum 1–4; and V1–V4, Vodka 1–4.

A total of 879 VOCs (661 identified VOCs) were detected in 24 distilled liquors (Table S1). Among the distilled liquors analyzed, C1 had the largest number of VOCs, with 466 compounds detected (344 identified VOCs). This was more than four-fold greater than V4, which had the lowest number of VOCs with 114. The amounts of VOCs detected in the whiskies, brandies, and rums were comparable, with more than 300 VOCs (Figure 1A). Among the VOCs detected, 159 were esters, 92 were alcohols, 105 were aldoketones, and 166 were benzene derivatives. A small number (23) of organic acids were also detected (Figure 1A).

Esters, as one of the most important classes of flavor compounds in distilled liquors, are commonly generated by esterification reactions between organic acids and alcohols during the fermentation and aging processes [21,22]. Esters had the highest total concentration among the VOCs in all distilled liquors assessed except for some vodka samples (V2, V3, and V4), in which benzene derivatives were more abundant (Figure 1B). In most Western liquors—such as whisky, brandy, and rum—octanoic acid ethyl ester had the highest ester concentration (ranging from 23 to 405 mg/L, none in R1), followed by decanoic acid ethyl ester (ranging from 21 to 70 mg/L, none in any whisky, R2, or R3). These two compounds constituted 8–48% of the total VOCs (10–48% in whiskies, 19–29% in brandies, and 8–47% in rums). The dominated esters were quite different in Chinese baijius. Lactic acid ethyl ester was the most abundant ester in Chinese soy sauce aroma type liquor (929 mg/L in C1 and 903 mg/L in C2), followed by acetic acid ethyl ester (771 mg/L in C1 and 851 mg/L in C2). Hexanoic acid ethyl ester had the highest concentration in Chinese strong aroma type liquor (772 mg/L in C3 and 643 mg/L in C4), followed by pentanoic acid ethyl ester (317 mg/L in C3 and 224 mg/L in C4). In Chinese light aroma type liquor, acetic acid ethyl ester was the dominant ester (250 mg/L in C5 and 167 mg/L in C6) followed by decanoic acid ethyl ester (125 mg/L in C5 and 165 mg/L in C6). The two of the most abundant esters constituted 23–29% of the total VOCs in Chinese baijius (Tables S1 and S2).

Alcohols and benzene derivatives alternated as the second most abundant VOCs in the distilled liquors assessed. Alcohols are produced by yeasts as by-products of reactions involving amino acids and carbohydrates [23]. The concentrations of alcohols differ significantly among distilled liquors ranging from 3 to more than 500 mg/L (account for 3–30%) (Figure 1C). Chinese soy sauce aroma type liquor had the highest concentration of alcohols (506 mg/L in C1 and 514 mg/L in C2), followed by Chinese strong aroma type liquor (417 mg/L in C3 and 390 mg/L in C4) (Figure 1B). However, R4 had the highest relative concentratons of alcohols accounting for 30% of the total VOCs, which was much higher than in Chinese baijius (7–11%), although their contents in R4 (217 mg/L) were lower than in Chinese baijius. Consistent with a previous study [23], 1-butanol, 3-methyl- was the major alcohol in most of the distilled liquors except for Chinese baijius, in which 1-hexanol was more abundant (Tables S1 and S2). 

Although the quantity of benzene derivatives were comparable with esters, their concentrations (40–372 mg/L) were much lower than esters (22–4044 mg/L) in the distilled liquors assessed (Figure 1A,B). Vodkas had the highest relative concentration of benzene derivatives (38–67%), which was much higher than those of other samples (5–26%) (Figure 1B). Benzene, 1,3-bis(1,1-dimethylethyl)- dominated the benzene derivatives accounting for 13–35% of total VOCs in vodkas. In contrast, benzaldehyde was the most abundant benzene derivative in other samples (Tables S1 and S2).

Aldoketones are other important flavor compounds in alcoholic beverages. Although aldoketones represented the third largest group of VOCs, their relative concentrations were generally low, ranging from 1% to 8% (Figure 1A,C). 

Organic acids were the least detected VOCs. This group of compounds had different distributions among the distilled liquors assessed. Middle-chain fatty acids, such as octanoic acid, nonanoic acid, and branched-chain 2,2-dimethylbutyric acid and heptanoic acid, 2-ethyl-, were the main organic acids in most Werstern liquors except for the vodkas, in which organic acids were barely detected. In Chinese baijius, octanoic acid and short chain fatty acid, such as acetic acid, butanoic acid, and hexanoic acid, were the predominated organic acids (Tables S1 and S2 ). 

### 2.2. Non-VOCs Detected by GC-TOFMS

**Figure 2:**
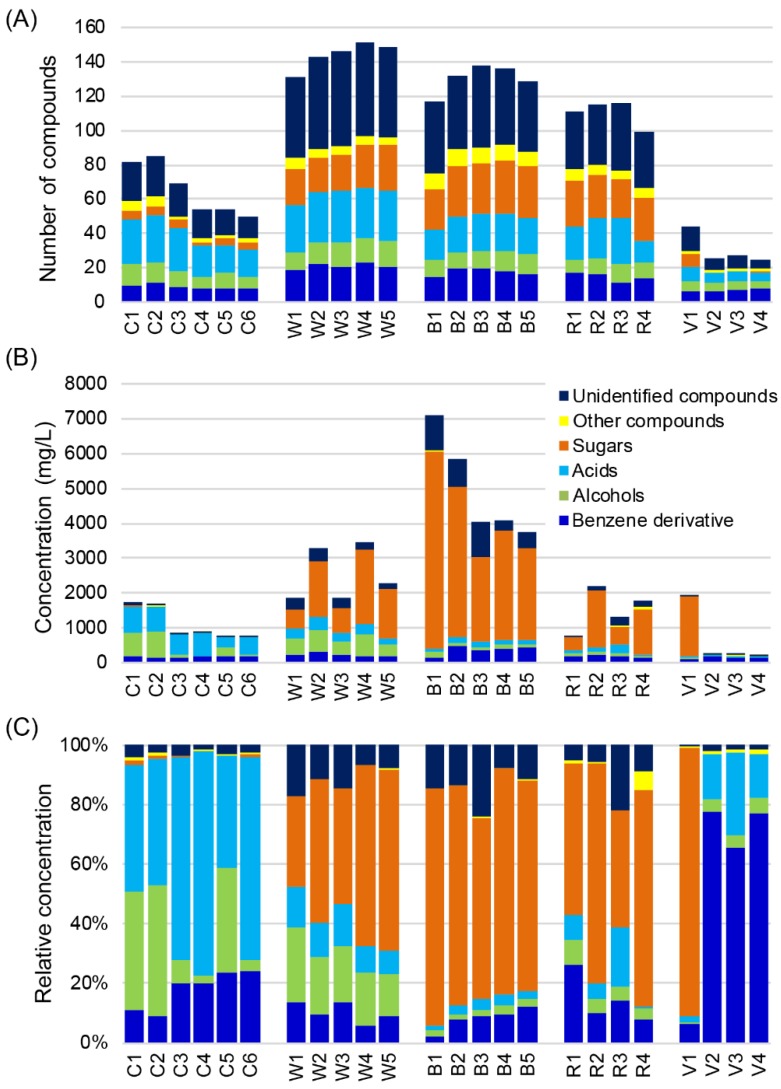
Non-volatile organic compounds (non-VOCs) in distilled liquors detected by GC-TOFMS. (**A**) Number of compounds in major classes of non-VOCs in 24 representative distilled liquors. (**B**) Contents of major classes of non-VOCs. (**C**) Relative concentrations of major classes of non-VOCs.

A total of 268 non-VOCs (160 identified non-VOCs) including organic acids, alcohols, sugars and benzene derivatives were detected by GC-TOFMS (Table S3). Most of these non-VOCs were detected in distilled liquors for the first time. Organic acids (46 compounds) were the most frequently detected non-VOCs, accounting for 17% of the total non-VOCs, followed by sugars (43 compounds), benzene derivative (30 compounds), and alcohols (27 compounds) (Figure 2A). These four classes of compounds constituted about 55% of the total non-VOCs detected. Esters, the dominating VOCs detected by HS-SPME-GC-TOFMS, were barely detected. 

The contents of non-volatile acids in Chinese baijius (283–727 mg/L) were generaly higher than in Western liquors (16–378 mg/L) (Figure 2B). Lactic acid was the major non-volatile acid in Chinese baijius (125–484 mg/L) accounting for 7–53% of the total non-VOCs. However, lactic acid was detected in only small amounts in Western liquors (1–44 mg/L). 

Sugars only comprised about less than 2% of the total non-volatile profile in Chinese baijius, but were the major non-VOCs in Western liquors, comprising from 30% to 90% of total non-VOCs except for some vodkas (V2 and V3) in which no sugar was detected (Figure 2C). Six-carbon (C6)-derived sugars, such as fructose and mannose, dominated the non-VOCs in Western liquors. Fructose accounted for more than 9% of the total non-VOCs in Western liquors, with the highest abundance (56%) found in B1 (Table S3 and Table S4).

The non-volatile alcohols detected were mainly polyols. Glycerol was the main non-volatile alcohol with content ranging from 9 to 553.2 mg/L in the Western liquors assessed. In contrast, the distribution of alcohols was more balanced in Chinese baijius. For example, in addition to glycerol, 2-methyl-1,3-propanediol and 2,3-butandiol were the main non-volatile alcohols, with 2,3-butandiol accounting for more than 50% of the total non-volatile alcohols in Chinese soy sauce aroma type liquor (Table S3 and Table S4). 

Although the contents of benzene derivatives were stable at 123–468 mg/L in the distilled liquors assessed, their relative concentration varied considerably, constituting 2–78% of total non-VOCs (Figure 2B,C). Benzene, ethyl, and benzene,1,4-dimethyl- were the most common benzene derivatives (Tables S3 and S4).

### 2.3. Characterization of Differences and Similarities of Chinese Baijius and Western Liquors by PCA and Correlation Network

In addition to the direct comparisons of VOCs and non-VOCs in Chinese baijius and Western liquors, we used more holistic analytical methods such as principal component analysis (PCA) and correlation network analysis to evaluate systemic differences amongst the samples.

**Figure 3:**
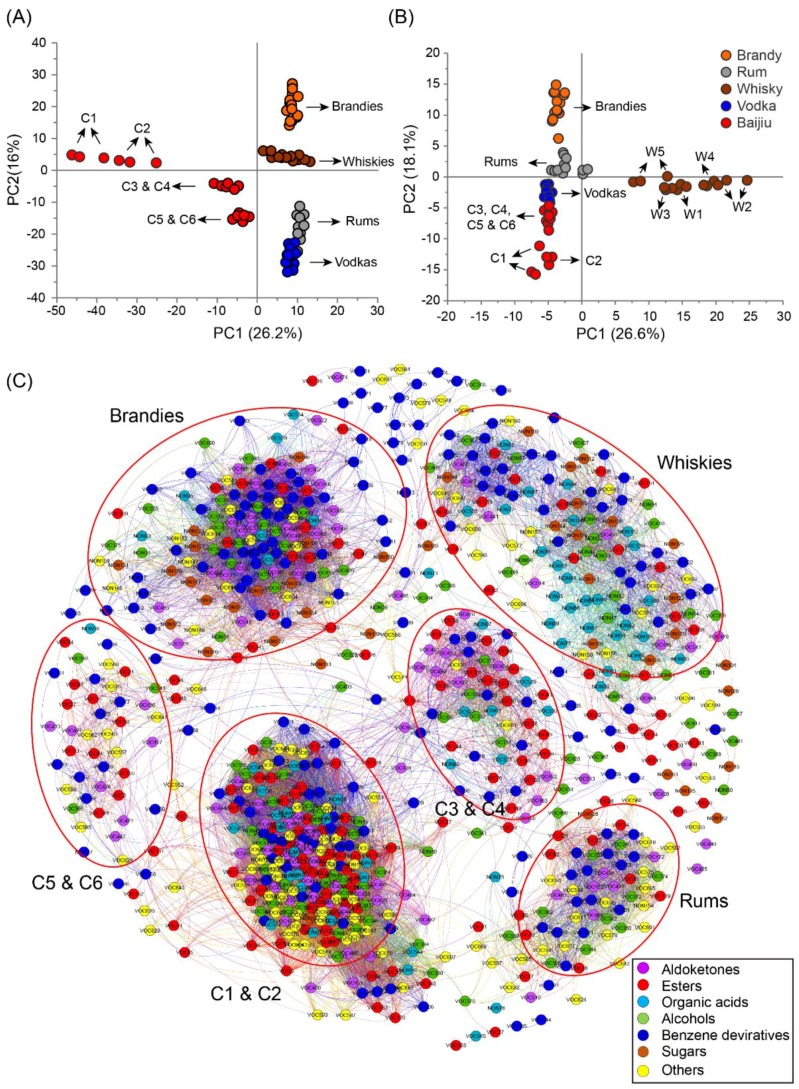
Principal component analysis (PCA) and correlation network analysis of 24 distilled liquors, including 6 Chinese baijius and 18 Western liquors. (**A**) Plot of PCA scores of VOCs detected by HS-SPME-GC-TOFMS (R2X = 0.962, Q2 = 0.863). (**B**) Plot of PCA scores of non-VOCs detected by GC-TOFMS (R2X = 0.902, Q2 = 0.745). (**C**) Network analysis clustering 777 significantly (FDR < 0.05) correlated compounds into six groups.

PCA, an unsupervised model that considers all variables, clusters or separates samples by reducing the dimensionality of variables. The first 10 principal components (PCs) of the VOCs-based PCA model described more than 80% of the data, with the first two PCs representing more than 42% of the variance in the data. The Chinese baijius were on the left of PC1 and all the Western liquors were located on the right of PC1. The first eight PCs of the non-VOCs-based PCA model described more than 80% of the data, with the first two representing more than 44% of the variance in the data. The Chinese baijius and vodkas were located on the lower left of PC1 and the rest of the Western liquors were distributed on the right of PC1 or above PC2. In both scores plots, Chinese baijius could be seperated from the Western liquors, suggesting their compositional differences (Figure 3A,B). Similar result was also obtained through heatmap analysis by conbining VOCs and non-VOCs (Figure S1).

Unsupervised PCA is superior for observing the similarities and differences between samples. However, PCA inevitably causes data loss during dimensionality reduction. Although the PCA loading plot has the potential to find variables that contribute to the the separation of samples, it also ignores important informations when there is a large number of variables (Figure S2). In addition, PCA analysis cannot reflect the correlations of variables that explain certain biological significance. Network analysis can demonstrate the relationship between variables. Network analysis identifies the co-occurrence of variables based on their correlation. When setting the threshold of the Pearson correlation coefficient at 0.7 (*ρ* > |0.7|), a total of 777 variables and 24,081 pairs of correlations were obtained. The Fruchterman–Reingold algorithm can clearly distinguish the structural information of the network without affecting the modular properties (such as density and average clustering coefficient) of the network. Compared with the network derived from other algorithms, such as Force Atlas, OpenOrd, Yifan Hu etc., the Fruchterman–Reingold-algorithm-based networks have better network topology and the nodes do not overlap. Hence, the Fruchterman–Reingold algorithm was used in our network. In the Pearson correlation-based network, we noticed that variables were clustered into six groups (Figure 3C). By extracting the clustered compounds, we found these compounds were exclusively detected in a certain type of liquor or their concentrations were higher than that of other types of liquors. Volatile benzene derivatives (e.g., ethanone, 1-(4-methylphenyl)-, benzoic acid, 2-hydroxy- ethyl ester, benzoic acid, pentyl ester, benzyl nitrile, naphthalene, 1,7-dimethyl-, 2-isobutoxyethyl benzoate, and dibenzofuran) and aldoketones (e.g., α-campholenal, 5-methyl-2-phenyl-2-hexenal, and 2,5-pyrrolidinedione), which were exclusively detected in brandies, were strongly linked and clustered on the top left of the network. The top right of the network mainly contained characteristic non-volatile benzene derivatives (e.g., homovanillyl alcohol and scopoletin) and acids (e.g., citric acid, α-Ketoglutaric acid, and glycolic acid) found in whiskies. Benzene derivatives (e.g., indan, 1-methyl-, benzofuran, 2-methyl-, benzoic acid, 2-methylpropyl ester, and phenol,2,4-dimethyl-), which were exclusively detected or had relative high concentration in rums, were clustered on the down right of the network. Volatile esters and benzene derivatives (e.g., butanoic acid, 3-methyl-, butyl ester, propanoic acid, 2-hydroxy-, 2-methylpropyl ester, octanoic acid, 3-methylbutyl ester, benzene, ethynyl-, 1-butanone, 1-phenyl-, and phenol, 2-ethyl-6-methyl-) were mainly clustered on the left of the network, which were exclusively found in Chinese light aroma type liquors (C5 and C6). Esters (e.g., hexanoic acid ethyl ester, ethyl 5-methylhexanoate, nonanoic acid ethyl ester, butyl octanoate, etc.) that were exclusively detected or had relatively high concentrations in Chinese strong aroma type liquors (C3 and C4) clustered in the middle right of the network. The characteristic compounds (e.g., propanoic acid, butyl ester, pentyl 2-methylbutanoate, butyl decanoate, benzofuran, benzeneacetic acid, butyl ester, 2-furanmethanol, 2H-pyran-2-one, tetrahydro-6-methyl-,4-acetyl-3-methylpyrazole, and 2,6-diethoxytetrahydropyran), found in Chinese soy sause aroma type liquors (C1 and C2), clustered in the bottom left of the network.

**Figure 4:**
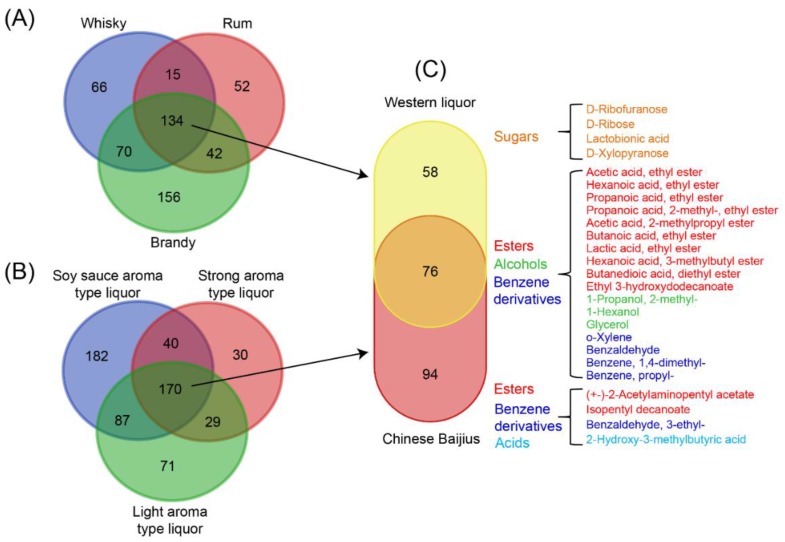
Venn diagram showing the similarities and differences of chemical composition between Chinese baijius and Western liquors. Number of unique and common compounds in (**A**) Western liquors and (**B**) Chinses baijius, and (**C**) the number of unique and common compounds between Western liquors and Chinese baijius. The listed compounds were distinct/shared compounds in Chinese baijius and Western liquors with concentrations more than 1 mg/L in most samples. Information about concentrations of the compounds in this figure can be found in Supplementary Tables.

In order to more intuitively illustrate the conpositional difference and similarity between Chinese baijius and Western liquors, we constructed Venn diagrams with all the identified compounds (Figure 4). Vodkas were not included because they only contained small amounts of compounds and these compounds did not cluster in the co-occurrence networks. After extracting the common compounds from each kind of distilled liquor (Figure S3), we observed that whiskies, brandies, and rums had 134 compounds in common (Figure 4A), and Chinese baijius had 170 compounds in common (Figure 4B). When comparing the common compounds between Western liquors and Chinese baijius, only 76 overlapped (Figure 4C, Table S5). The compounds shared by both Western liquors and Chinese baijius were mainly ethyl esters (e.g., acetic acid ethyl ester, propanoic acid ethyl ester, butanoic acid ethyl ester, hexanoic acid ethyl ester, lactic acid ethyl ester, etc.), a few benzene derivatives (e.g., o-xylene and benzaldehyde), and alcohols (e.g., 1-propanol, 2-methyl-, 1-hexanol and glycerol etc.), suggesting the important roles of these compounds in the flavor and mouthfeel of distilled liquor. The unique compounds in Chinese baijius were mainly esters formed by fatty alcohols such as hexanoic acid, hexyl ester, isopentyl heptanoate, and isopentyl decanoate. Sugars, such as lactobionic acid, d-xylopyranose, d-ribose, and d-(-)-ribofuranose, were exclusively detected Western liquors (Figure 4C). This indicates that these esters and sugars can be used for differentiating Chinese baijius and the Western liquors.

## 3. Discussion

In this study, we analyzed the compositions of 24 representative distilled liquors from around the world. In addition to detecting the VOCs via conventional HS-SPME-GC-TOFMS, we applied a classical metabonomics approach to detect non-VOCs through derivatization, as non-VOCs are difficult to directly analyze using GC–MS. Derivatization can increase the volatility and thermal stability of the analytes [24], and produce better MS properties and more favorable diagnostic fragmentation patterns for structure identification [25]. This methodology has been applied to study the chemical compositions of various food products—including fruits [26], sake [27], honey [28], and roselle [29]—and was proven to be efficient for the detection of non-VOCs. Our results suggest that derivatization-based GC-TOFMS is highly complementary to the detection range of HS-SPME-GC-TOFMS and both methods are indispensable for studying the complete composition of distilled liquors. 

**Figure 5:**
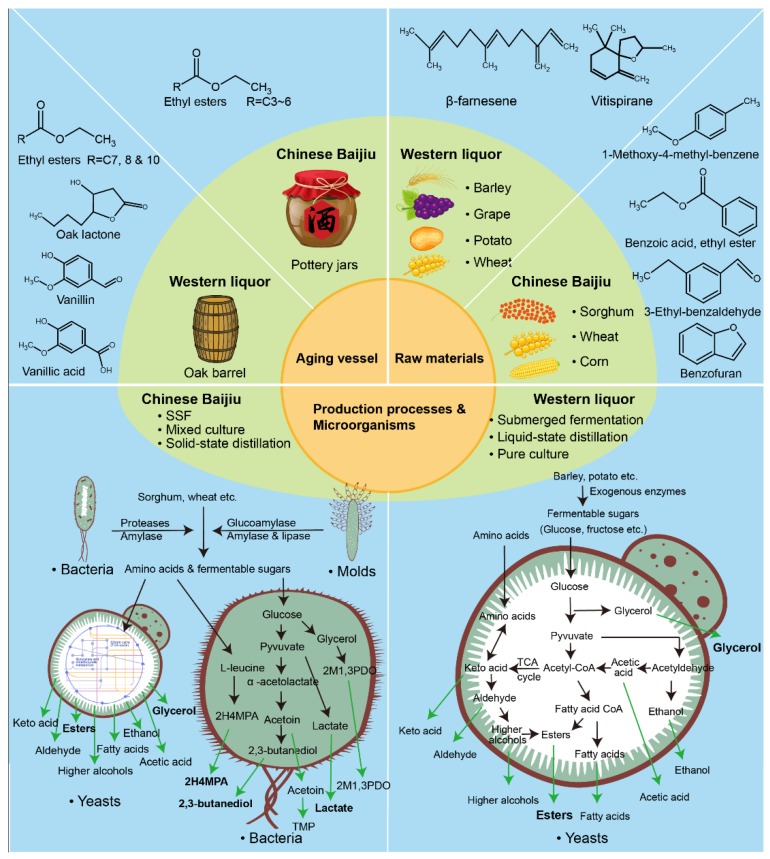
Factors that contribute to the compositional difference between Chinese baijius and Western liquors, and representative compounds associated with each factor. In the orange core circle are the major contributing factors: raw materials, production processes, microorganisms, and aging vessel. In the middle green ring are the corresponding elements of the factors. The blue periphery ring are key compounds influenced by the core factors. The model of the production processes and microorganisms briefly describes the biochemical production pathways of a few major compounds. C7, 8 and10 represents R contains C7, C8, and C10 linear chain length, respectively; C2–C6 represent R containing C2–C6 linear chain length, respectively. SSF: simultaneous saccharification and fermentation; 2H4MPA: 2-hydroxy-4-methyl-pentanoic acid; 2M1, 3PDO: 2-methyl-1,3-propanediol; TMP: tetramethyl-pyrazine. Information about concentration of the compounds in this figure can be found in Supplementary Materials.

Our results highlight that both VOCs and non-VOCs are notably different between Chinese baijius and Western liquors. These differences may be influenced by many factors including raw materials, production processes, microorganisms, and aging vessels (Figure 5). Raw materials can play direct or indirect roles. Some ingredients of raw material can be directly incorporated into distilled liquors. For example, β-famesene and vitispirane, which were exclusively detected in the brandies, are important aroma components of grape and these compounds are commonly detected in wine [30]. Some raw materials play indirect roles by being degraded by microorganisms. Chinese baijius are produced from sorghum or a mixture of wheat, corn, and sorghum. These protein-rich raw materials can produce some sulfur compounds by degrading sulfur-containing amino acids [31]. Our previous study using metatranscriptomic analysis revealed that, during the alcoholic fermentation of Chinese baijiu, *Lactobacillus* was active in the methyl cycle, which recycles methionine, which is the precursor of the two sulfur compounds [32]. Some raw materials can also provide benzene derivatives from the degradation of lignin, which is abundant in the hulls of these grains [31]. Benzene derivatives may exist in soluble and bound forms in plants. Bound phenolics are formed by the combination of soluble phenolics and cell wall macromolecules (e.g., polysaccharides and proteins) as a component of plant cell walls [33]. It has been reported that fermentation increases the total phenolics content (TPC). Because soluble phenolics can be released from bound phenolics during the fermentation process [34]. This may be related to the microbe-mediated decomposition of cell wall components and subsequent release of bound phenolics, since many microbes possess various enzymes—such as cellulase, feruloyl esterase, glucosidase, xylanase, pectinase, and proteinases—which can degrade the cell wall matrix [35].

Production processes and brewing microorganisms are the major factors contributing to the compositional differences between Chinese baijius and Western liquors. The high concentration of non-volatile acids in Chinese baijius is inseparable from the unique production process of Chinese baijius [3]. The fermentation process of Chinese baijiu uses unique simultaneous saccharification and fermentation (SSF). A large proportion of lactic acid bacteria (LAB) and *Clostridium* sp. are involved. These strains efficiently produce lactic acid, butanoic acid, hexanoic acid, and corresponding ethyl esters [36]. Some LAB also use L-leucine to produce 2-hydroxy-4-methyl-pentanoic acid [37], which was exclusively detected in Chinese baijius in considerable amounts. *Bacillus* sp. including *B. subtilis*, *B. licheniformis*, and *B. amyloliquefaciens* are involved in Daqu production and the following stacking fermentation. *Bacillus* sp. are not only known as the producer of various hydrolases, but also as efficient producers of 2,3-butanediol [38,39]. *B. subtilis* has been reported to produce tetramethyl-pyrazine [40], which was exclusively detected in Chinese baijius. In contrast, *Saccharomyces cerevisiae*—known to produce ethyl octanoate, ethyl decanoate, glycerol, and minor higher alcohols, fatty acids, and aldoketones [23,41]—was the main microorganism in the production of Western liquors.

Aging vessels is another important factor. Many important flavor compounds come from the aging vessels, such as oak lactone (also known as 2(3H)-furanone, 5-butyldihydro-4-methyl-, cis-), vanillin, and vanillic acid. These compounds originally resided in oak barrels in which most Western liquors were aged [42], whereas the Chinese baijius were aged in pottery jars. Hence, these compounds can be only detected in Western liquors (Table S1 and S2). In addition, artificial factor contribute to the compositional difference between Chinese baijius and Western liquors. Sugars, the main non-volatile compound in Western liquors, are commonly added artificially to aged Western liquors such as brandy [11], rum [43], and whisky [44], giving them an amber coloration that is attractive to consumers. The artificially added sugar in distilled liquors is collectively called caramel. The chemical composition of caramel is complex due to the large number of substances produced as a result of pyrolysis of carbohydrates, such as sucrose, glucose, or starch. Caramel has been reported to be rich in advanced glycation end products (AGEs). AGEs are blamed for increasing insulin resistance and inflammation [45]. 

However, many bioactive compounds including aldoketone, pyrazine, alcohols, fatty acids, and ethyl esters were observed in distilled liquor [46] and most of them were detected in this study. Additionally, many of the detected benzene derivatives—such as benzofuran, benzoic acid ethyl ester, benzene acetic acid ethyl ester, benzene propanoic acid ethyl ester, and phenylethyl alcohol—are known to be bioactive. An in vitro study reported that a honey that was abundant with these benzene derivatives exhibited effective antibacterial activity, especially for Gram-negative bacteria [28]. The progress of alcoholic liver disease has shown that alcohol administration can cause overgrowth of intestinal Gram-negative bacteria and increase the burden in the intestine, leading to leaky gut. Lipopolysaccharide (LPS), a critical component of the outer membrane of Gram-negative bacteria, then translocates from the gut lumen to the liver and leads to alcoholic hepatitis [47]. Benzene derivatives in distilled liquors may be able to play a role in mitigating the enteric dysbiosis caused by ethanol. Moreover, many heterocyclic compounds, such as furans, pyrroles, thiophenes, and thiazoles, that have been detected in Chinese liquors by using more advanced comprehensive two-dimensional GC with time-of-flight mass spectrometry (GC × GC-TOFMS) [46,48] are bioactive as well. Heterocyclic compounds widely exist in coffee and have certain levels of antioxidative activity [49]. In vivo, ethanol is mainly metabolized by alcohol dehydrogenase into acetaldehyde, which is responsible for the generation of reactive oxygen species (ROS). ROS can cause oxidative stress and further lead to liver injury [50]. Although this activity is not as strong as that of the synthetic antioxidant butylated hydroxytoluene (BHT), the combined activity of large numbers of these heterocyclic compounds might be comparable to those of known antioxidants [49]. Therefore, the heterocyclic compounds in distilled liquor may mitigate oxidative damage caused by ethanol. Further research could design animal models to understand whether the presumed bioactive compounds in distilled liquor really play specific roles by exploration of gut microbiota and host metabolism.

## 4. Conclusions

We analyzed the complete chemical compositions of 24 representative distilled liquors from around the world using GC–MS-based metabolomics approaches. The compositional differences and similarities between Chinese baijius and Western liquors were revealed by multivariate statistics and correlation network analysis. Many esters, benzene derivatives, and alcohols were shared by most of the tested distilled liquors, suggesting their important contribution to the common flavor of distilled liquors. Certain sugars and esters formed by fatty alcoholare potential indicators that can be used for differentiating Chinese baijius and Western liquors. Factors including raw materials, production processes, microorganisms, and aging vessels could all contribute to the differences between Chinese baijius and Western liquors. To the best of our knowledge, this is the first study that comprehensively analyzed both VOCs and non-VOCs in distilled liquors and systematically compared the compositional differences and similarities between typical Chinese baijius and Western liquors. This study provides a new perspective for analyzing the chemical composition of distilled liquors and improves our molecular understanding of this plant-based fermented product.

## 5. Materials and Methods 

### 5.1. Reagents

n-hexane was purchased from Merck (Darmstadt, Germany). Anhydrous sodium sulphate was analytical grade and obtained from the China National Pharmaceutical Group Corporation (Shanghai, China). N,O-bis-(trimethylsilyl)-trifluoroacetamide with 1% trimethylchlorosilane (BSTFA + 1% TMCS) used for derivatization was purchased from Thermo Scientific (Bellefonte, PA, USA). Pyridine, methoxyamine hydrochloride, n-alkanes of C4–C30, and fatty acid methyl esters (FAME markers) of C8, C9, C10, C12, C14, C16, C18, C20, C22, C24, C26, C28, and C30 linear chain lengths were purchased from Sigma-Aldrich (St. Louis, MO, USA). l-menthol and l-2-chlorophenylalanine (Sigma-Aldrich, Shanghai, China) were used as internal standards.

### 5.2. Distilled Liquors

A total of 24 distilled liquors were used in this study including 6 Chinese baijius and 18 Western liquors (Table 1). Distilled liquors were purchased from the local market or spplied by distilleries.

### 5.3. Sample Preparation

#### 5.3.1. VOC Extraction 

The sample preparation and HS-SPME technique were performed according to the methods described previously with minor changes [51]. Each liquor sample was diluted with deionized water to a final concentration of 10% (*v*/*v*) ethanol. A total of 10 mL diluted solution saturated with sodium chloride was placed into a 20 mL screw-capped vial, and spiked with 10 μL internal standard L-menthol to a final concentration of 15.625 mg/L. Then, the vial was tightly capped with a silicon septum. This sample was equilibrated at 50 °C in a thermostatic water bath for 15 min prior to analysis. After equilibration, a 2 cm fiber coated with 50/30 μm divinylbenzene (DVB)/carboxen (CAR)/polydimethylsiloxane (PDMS; Supelco, Bellefonte, PA, USA) was exposed to the headspace of the vial for 30 min at the same temperature. The fiber was then inserted into the injection port of GC (250 °C) for 5 min to desorb the analytes. Each sample was analyzed in triplicate.

#### 5.3.2. Extraction, Oximation, and Derivatization of Non-VOCs 

Three milliliters of each distilled liquor were spiked with 10 μL internal standard l-2-chlorophenylalanine to a final concentration of 16.3 mg/L and vortexed for 10 s, and then extracted with equal volumes of n-hexane thrice. After extraction, 1 mL of the aqueous phase was collected into GC–MS vials and then freeze-dried in a vacuum centrifuge. The dried residue was then resuspended in 50 μL of methoxyamine in pyridine (15 mg/mL) and incubated in a vortex mixer (Fisher Scientific, Springfield, NJ, USA) for 90 min at 30 °C. The resulting solution was derivatized with 50 μL BSTFA spiked with FAME at 70 °C for 60 min. Each sample was analyzed in triplicate. Replication of each distillate in this study included different bottles from the same vendor.

### 5.4. Metabolomics Analyses

Both VOC and non-VOC detection was performed on an Agilent 7890B (Santa Clara, CA, USA) gas chromatograph equipped with a Pegasus HT time-of-flight (TOF) mass spectrometer (Leco Corporation, St. Joseph, MI, USA).

For the detection of VOCs, samples were analyzed on a DB-FFAP column (60 m × 0.25 mm i.d., 0.25 μm film thickness, Agilent JandW Scientific, Folsom, CA, USA). The detector and injector temperature was 250 °C. The oven temperature was held at 45 °C for 3 min, and then raised to 150 °C at a rate of 4 °C per min, and held for 2 min, then raised to 200 °C at a rate of 6 °C per min, then raised to 230 °C in 10 min, and held for a final 15 min. The column carrier gas was helium at a constant flow rate of 1 mL/min. Splitless injection mode was used. The temperature of the ion source was 230 °C, and the electron impact mass spectra were recorded at 70 eV ionization energy. The GC–MS analysis was carried out in the scanning mode (SCAN) in the 35–400 aμ mass range, and the acquisition rate was 100 spectrum/s in the TOFMS setting.

For the detection of non-VOCs, each 1-μL aliquot of the derivatized solution was injected in split mode (split ratio 1:10). Separation was achieved on a DB-5 ms capillary column (30 m × 250 μm i.d., 0.25 μm film thickness, Agilent JandW Scientific, Folsom, CA, USA). Helium was used as the carrier gas at a constant flow rate of 1.0 mL/min. The temperatures of injection, transfer interface, and ion source were set to 250, 250, and 230 °C, respectively. The GC temperature programming was set to 2 min of isothermal heating at 50 °C, followed by 6 °C/min oven temperature ramps to 230 °C, and a final 15 min maintenance at 230 °C. MS measurements were recorded with electron impact ionization (70 eV) in full-scan mode (*m/z* 30–600), and the acquisition rate was 20 spectrum/second in the TOFMS setting. 

To ensure the stability of the system over the different sequences and provide confidence in our data, we completed the following. Before testing, a tuning solution was used to ensure the stability of the fragment ion. During testing, we ensured the stability of chromatography and column by monitoring the retention index of n-alkane or fatty acid methyl ester. We also extracted the intensity of methyl tridecanoate and evaluated its intensity range (usually ranged from 80% to 120% of average intensity). In addition, a mixture solution with all the sample extracts was used as quality control (QC) samples. QC samples were injected (after a blank) every nine samples in the batch. The samples were measured alternately, such as “C1–R1–W1…”, to minimize or eliminate systematic analytical deviations. All samples were analyzed in triplicate and the results were averaged. To ensure the stability and the repeatability of the equipment, we performed PCA using all the sample data along with the QCs (Figure S4).

### 5.5. GC–MS Data Pretreatment, Compound Identification, and Quantification

The operation procedures for GC–MS data pretreatment were developed by our laboratory [52]. The acquired MS files from GC-TOFMS analysis were exported in NetCDF format by ChromaTOF software (v3.30, Leco Co., CA, USA). CDF files were extracted using custom scripts (revised MATLAB toolbox hierarchical multivariate curve resolution (HMCR), developed by Par Jonsson, et al. [53]) in MATLAB 7.0 (The MathWorks, Inc., MA, USA) for data pretreatment procedures such as baseline correction, de-noising, smoothing, alignment, time-window splitting, and multivariate curve resolution (based on the multivariate curve resolution algorithm). The optimized parameters were as follows: signal-to-noise (S/N) = 30, baseline offset = 0.5, and peak width = 2. The resulting three-dimensional dataset included sample information, peak retention time, and peak intensities. Retention index (RI) calibration and mass spectral deconvolution were performed with ChromaTOF™ software (v3.30, Leco Co., CA, USA). Commercial mass spectral databases such as Wiley 9, mainlib, and replib were fully integrated with the ChromaTOF™ deconvolution software and were chosen for mass spectral comparison. The selected VOCs and non-VOCs were positively identified by comparing their mass spectral data and RI values with those of authentic standard compounds. The RIs of VOCs were calculated using n-alkanes C4–C30 as external references. The RIs of non-VOCs were calculated using the FAME of C8, C9, C10, C12, C14, C16, C18, C20, C22, C24, C26, C28, and C30 linear chain lengths [54]. Mass spectral matching was manually supervised and matches were accepted with thresholds of match >800 (with maximum match equal to 1000) and reconfirmed by RI values (± 25) when available. All identified compounds met level 2 or above of the metabolite identification set by the Chemical Analysis Working Group (CAWG) Metabolomics Standards Initiative (MSI) [55]. Compounds were quantified using the method proposed and validated by Gao et al. [56].

### 5.6. Statistical Analysis

The data matrices were introduced into SIMCA-P+ 13.0 (Umetrics, Umeå, Sweden) software for PCA. Prior to PCA, all variables obtained were mean-centered and scaled to the pareto variance. PCA is an unsupervised technique that reduces the dimensionality of the original data matrix while retaining the maximum amount of variability. Therefore, PCA can explain the differences between distilled liquors by means of factors obtained from the datasets and, simultaneously, determine which variables contribute most to such differences. Correlative network was visualized with Gephi (Version 0.9.1; available at: https://gephi.org) [57] using the classic Fruchterman–Reingold algorithm. Before plotting in a correlative network, pairwise Pearson’s correlations between variables were computed using SPSS Version 22 (SPSS Inc., Chicago, IL, USA) and the significant correlations (FDR < 0.05) were retained. The FDR value was calculated by R (Version 3.2.2) with the fdrtool package.

## Figures and Tables

**Table 1:** Distilled liquors used in this study

	Type of Distillate	Abbreviation	Place of Origin	Raw Material
Chinese baijius	Soy sauce aroma type liquor	C1	Guizhou, China	Sorghum, wheat
	Soy sauce aroma type liquor	C2	Guizhou, China	Sorghum, wheat
	Strong aroma type liquor	C3	Sichuan, China	Sorghum, wheat, corn
	Strong aroma type liquor	C4	Anhui, China	Sorghum, wheat, corn
	Light aroma type liquor	C5	Shanxi, China	Sorghum, barley, pea
	Light aroma type liquor	C6	Beijing, China	Sorghum, barley, pea
Western liquors	Whisky	W1	Kentucky, America	Barley malt, corn, caramel
	Whisky	W2	Speyside, Scotland	Barley malt, caramel
	Whisky	W3	Ireland	Barley malt, corn, caramel
	Whisky	W4	Scotland	Barley malt, corn, caramel
	Whisky	W5	Scotland	Barley malt, caramel
	Brandy	B1	France	Grape, caramel
	Brandy	B2	France	Grape, caramel
	Brandy	B3	France	Grape, caramel
	Brandy	B4	France	Grape, caramel
	Brandy	B5	France	Grape, caramel
	Rum	R1	Puerto Rico	Sugar cane, caramel
	Rum	R2	U.S.	Sugar cane, caramel
	Rum	R3	France	Sugar cane, caramel
	Rum	R4	Cuba	Sugar cane, caramel
	Vodka	V1	Britain	Wheat, barley malt
	Vodka	V2	Sweden	Wheat
	Vodka	V3	France	Wheat
	Vodka	V4	Poland	Wheat, potato

## Supplementary Materials

The following are available online at http://www.mdpi.com/2218-1989/9/1/2/s1, Table S1: Volatile compounds and their relative concentration (%) in 24 distilled liquors detected by HS-SPME-GC-TOFMS, Table S2: Representative VOCs and their concentration (mg/L) in distilled liquors mentioned in the main text, Table S3: Non-volatile compounds and their relative concentration (%) in 24 distilled liquors detected by GC-TOFMS, Table S4: Representative Non-VOCs and their concentration (mg/L) in distilled liquors mentioned in the main text, Table S5: Compounds involved in Figure 4, Figure S1: Heatmap showing the clustering of the samples, Figure S2: PCA loadings of the (A) VOCs and (B) non-VOCs, Figure S3: Venn diagrams extract the common compounds from each kind of distilled liquor, Figure S4: The PCA plot of QCs and samples demonstrate the stability and the repeatability of the equipment.
