# Supplementary figures and images for: Compositional Differences and Similarities between Typical Chinese Baijiu and Western Liquor as Revealed by Mass Spectrometry-Based Metabolomics
Source: Metabolites. 2018 Dec 21;9(1):2. doi: 10.3390/metabo9010002 (PMC6358772; doi:10.3390/metabo9010002)

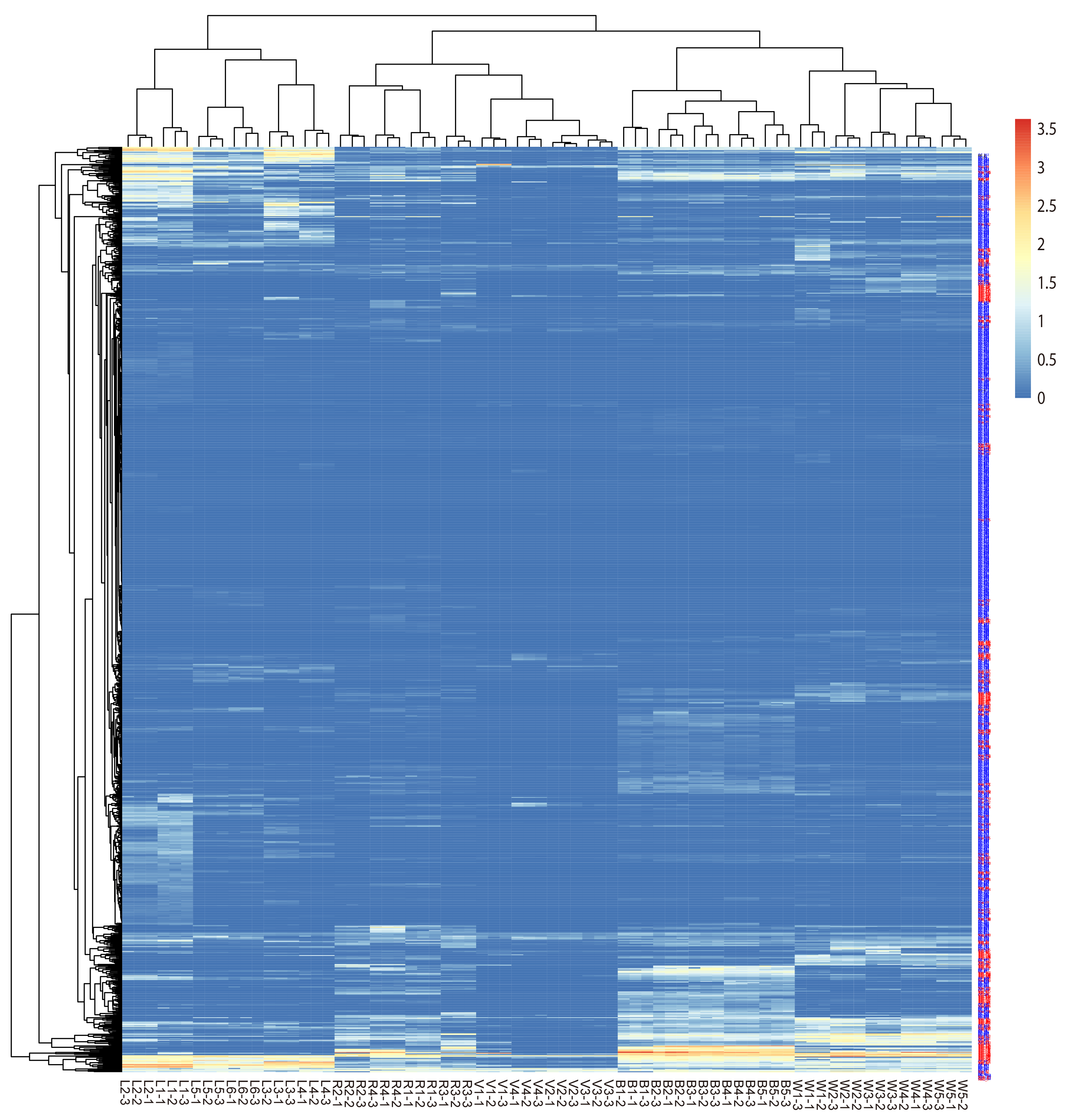

Supplement: Supplementary file 1 [file metabolites-09-00002-s001.zip › Figure S1.tif]

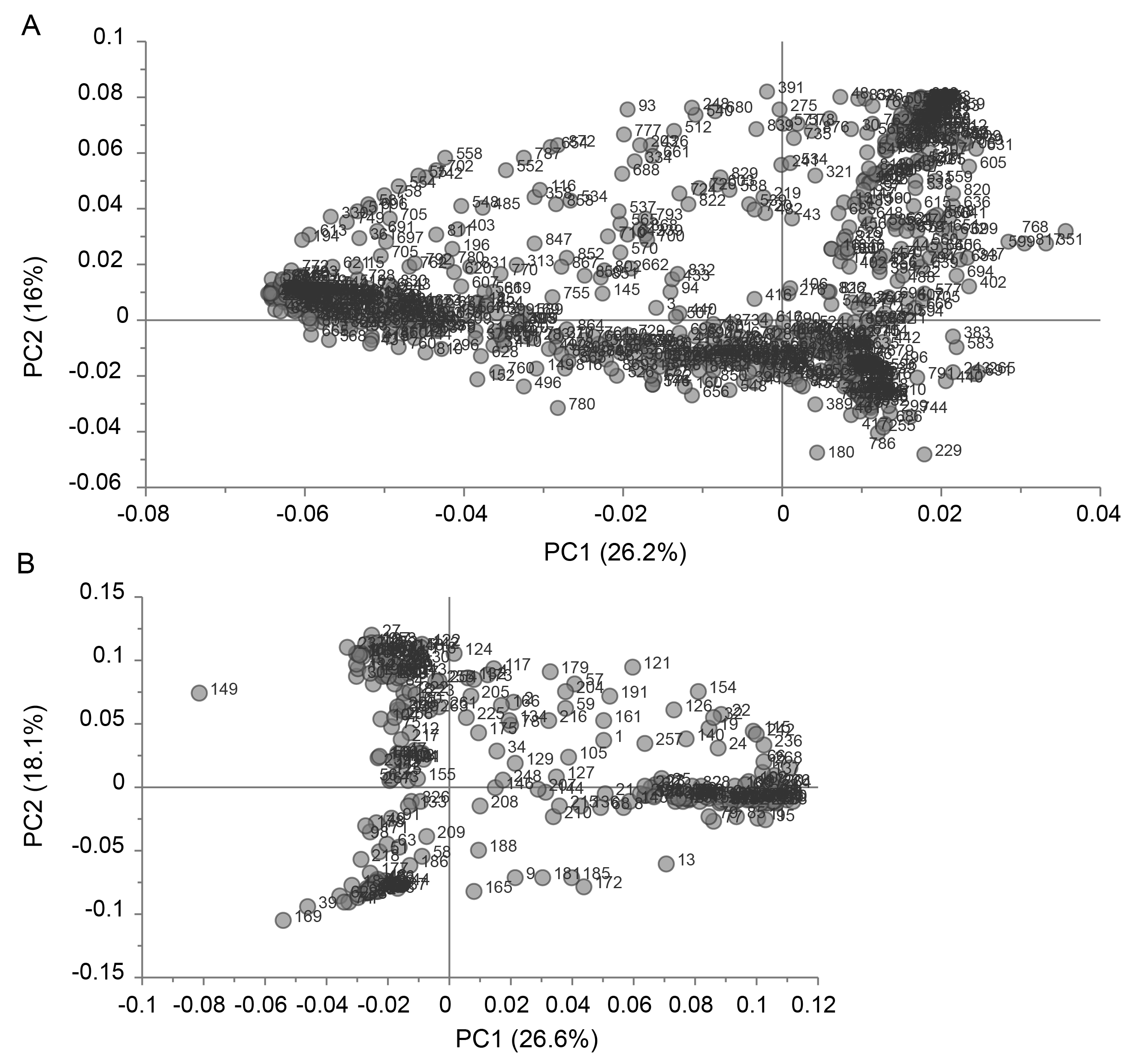

Supplement: Supplementary file 1 [file metabolites-09-00002-s001.zip › Figure S2.tif]

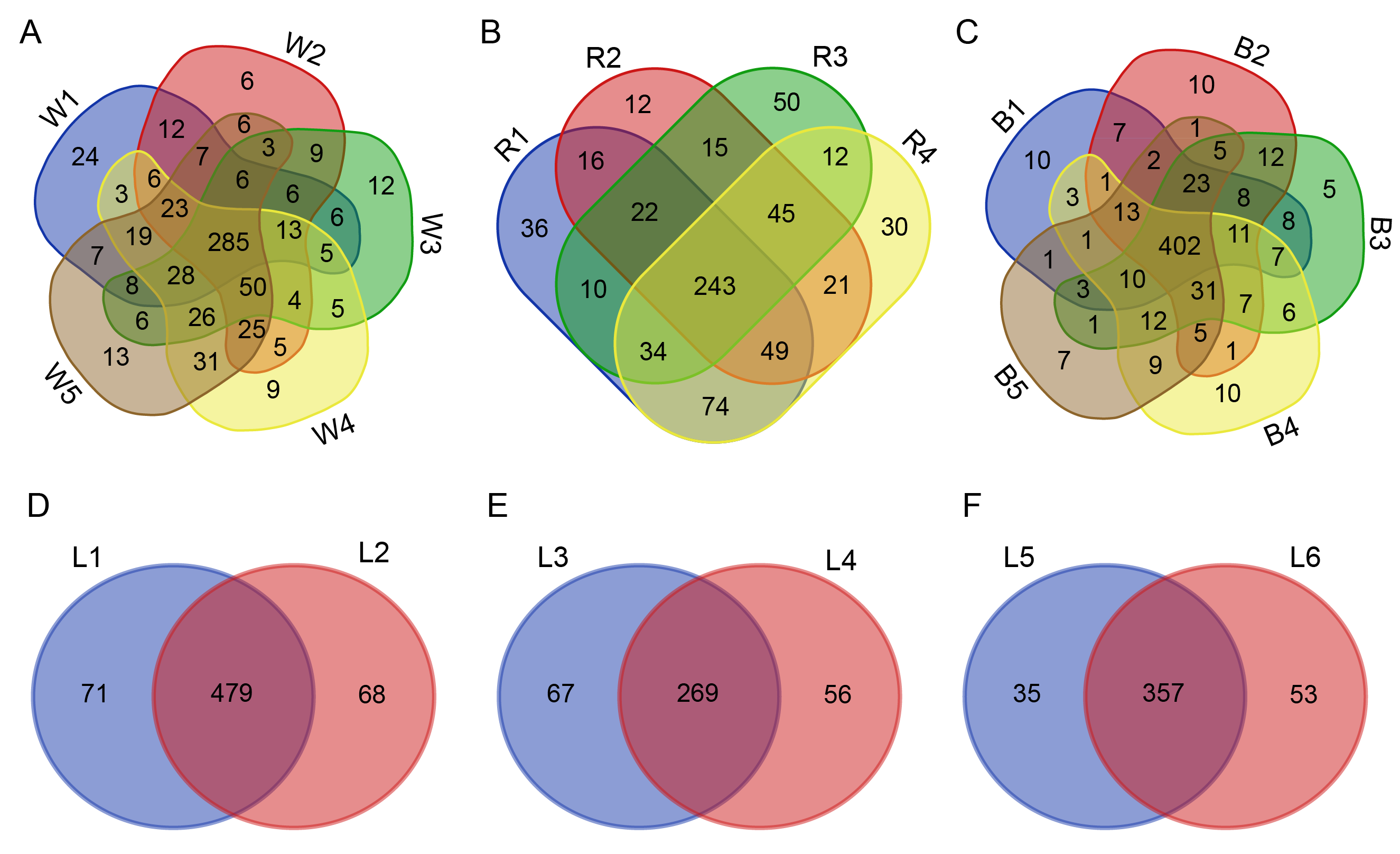

Supplement: Supplementary file 1 [file metabolites-09-00002-s001.zip › Figure S3.tif]

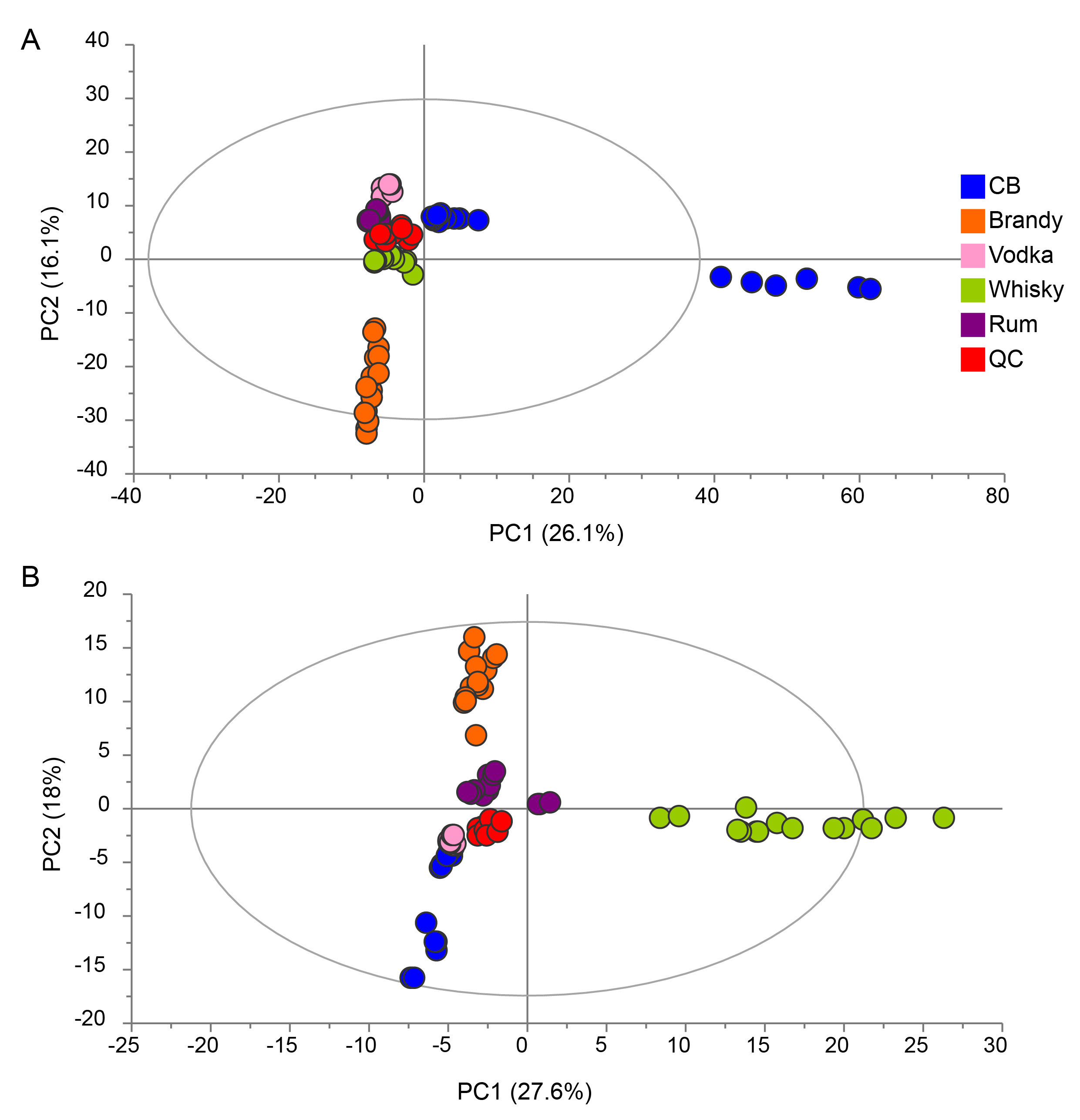

Supplement: Supplementary file 1 [file metabolites-09-00002-s001.zip › Figure S4.tif]
